# Fortuitous Discovery of Nutcracker Syndrome During Acute Appendicitis: A Case Report

**DOI:** 10.7759/cureus.32109

**Published:** 2022-12-01

**Authors:** El Mehdi Mniai, Yassamin Benhayoun Sadafyine, Amine Benfaida, Mohamed Mahi, Amal Rami

**Affiliations:** 1 Radiology, Mohammed VI Center for Research & Innovation, Cheikh Khalifa International University Hospital, Mohammed VI University of Health Sciences (UM6SS), Casablanca, MAR; 2 Gastroenterology, Mohammed VI Center for Research & Innovation, Cheikh Khalifa International University Hospital, Mohammed VI University of Health Sciences (UM6SS), Casablanca, MAR

**Keywords:** : pelvic pain, renal nutcracker syndrome, renal veins, hematuria, abdominal pain

## Abstract

Nutcracker syndrome (NCS) belongs to a group of rare vascular disorders. It refers to compression of the left renal vein (LRV) generally between the abdominal aorta (AA) and the superior mesenteric artery (SMA). It is one of the most unknown causes of chronic abdominal pain. Herein, we present the case of a young patient who came to the emergency department for acute abdominal pain. Patient's history revealed an uncharacterized chronic epigastric pain evolving for 13 years. The imaging showed acute appendicitis and NCS; the latter finding was the principal explanation for the patient’s chronic pain. We hope that the concise and synthetized structure of this case report will help physicians acquire the necessary reflexes to notice and diagnose this already underdiagnosed syndrome.

## Introduction

Nutcracker syndrome (NCS), also known as left renal vein (LRV) entrapment, belongs to a group of rare vascular disorders [1]. NCS encompasses all manifestations related to venous stasis induced by stricture of the left renal vein mostly between the abdominal aorta (AA) and the superior mesenteric artery (SMA) [2]. It was first described anatomically by Grant in 1937 as follows: "...the left renal vein, as it lies between the aorta and the superior mesenteric artery, resembles a walnut between the jaws of a nutcracker" [3]. NCS is one of the most unknown causes of chronic abdominal pain and can be a serious condition [4]. We report a case of a fortuitous discovery of NCS during an appendicular crisis in a patient reporting chronic epigastric pain.

## Case presentation

A 32-year-old woman presented to the emergency department for a history of acute pain in the right lower abdomen evolving for two days. She also complained of acute diarrhea, nausea and fever. After a thorough anamnesis, she reported a 13 years history of intermittent epigastric pain. The patient did not relate any episode of hematuria, dyspareunia or sensation of pelvic heaviness. She consulted at several structures before and received symptomatic treatment based on non-opioid analgesics. Nonetheless, no imaging was performed, the origin of the pain has never been explored and no organic cause was found. 

**Figure 1:**
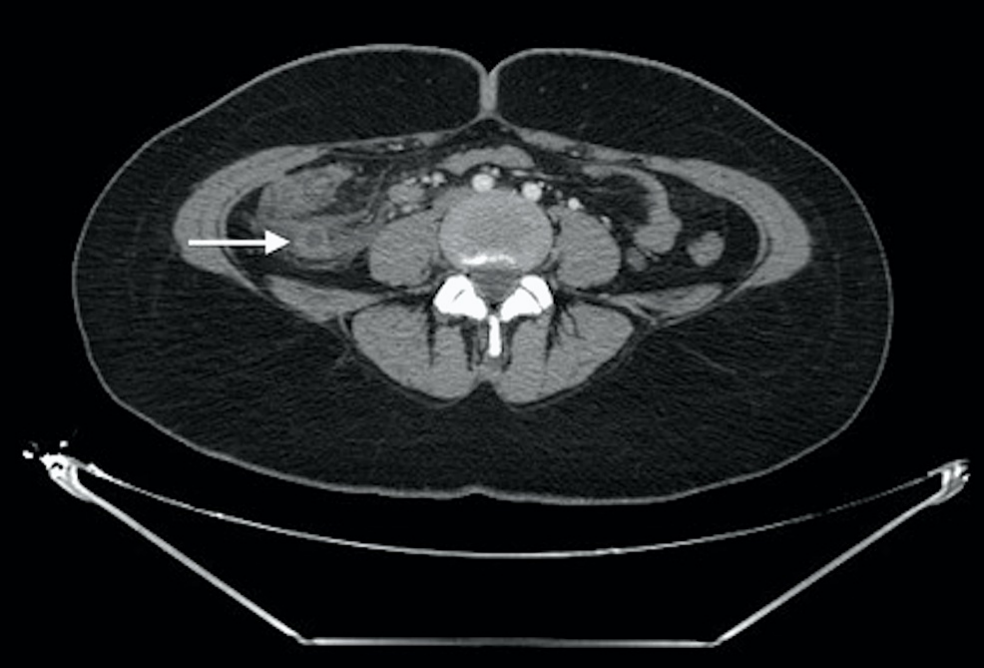
Axial CT scan showing swollen appendix surrounded by mesenteric fat infiltration and peritoneal effusion: Acute appendicitis (white arrow) CT: Computed tomography

Physical examination showed normal blood pressure of 125/70 mmHg, regular pulse of 79 beats/min, low-grade fever of 38.1 °C, and respiratory rate of 17 breaths/min. There was a tenderness in the right iliac fossa with positive McBurney sign. Laboratory assessment revealed negative ß-human chorionic gonadotropin (ß-HCG), hyperleukocytosis of 12 000, C-reactive protein (CRP) of 25, sterile cytobacteriological examination of urine (CBEU), and normal lipasemia. An abdominal and pelvic Computed Tomography (CT) scan after contrast injection showed a swollen appendix (9 cm thick) surrounded by mesenteric fat infiltration and peritoneal effusion (Figure 1). These CT scan features suggested acute appendicitis. The patient underwent laparoscopic appendectomy with no per- or postoperative complications.

**Figure 2:**
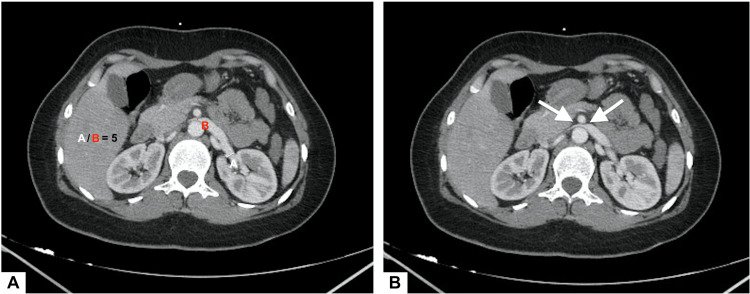
Axial CT angiography showing the compressive phenomenon A: Dilatation of the left renal vein with a ratio of hilar to aorto-mesenteric diameters > 4,9 (A/B = 5). B: White arrows indicating the narrowing of left renal vein segment trapped between the abdominal aorta and the superior mesenteric artery ("beak sign")

**Figure 3:**
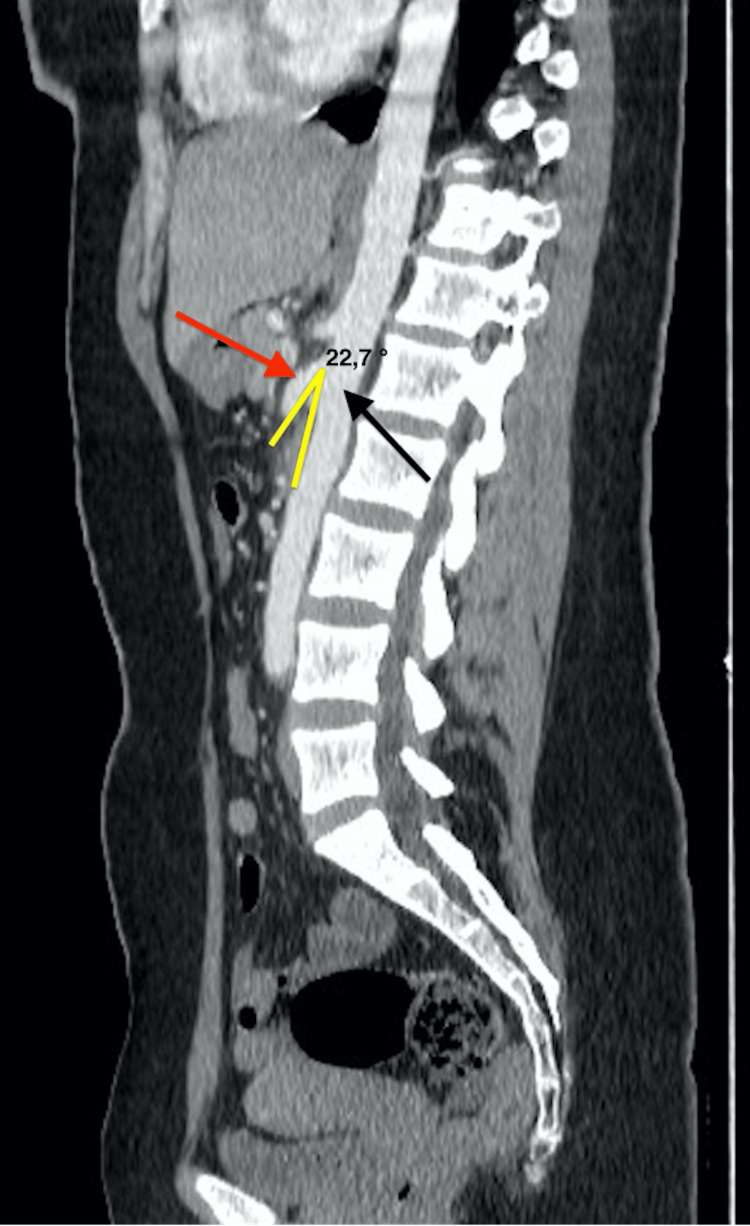
Sagittal CT angiography section showing an angulation between the abdominal aorta and the superior mesenteric artery of 22,7°. Black arrow: abdominal aorta Red arrow: superior mesenteric artery The angle between the two vessels is in yellow.

**Figure 4:**
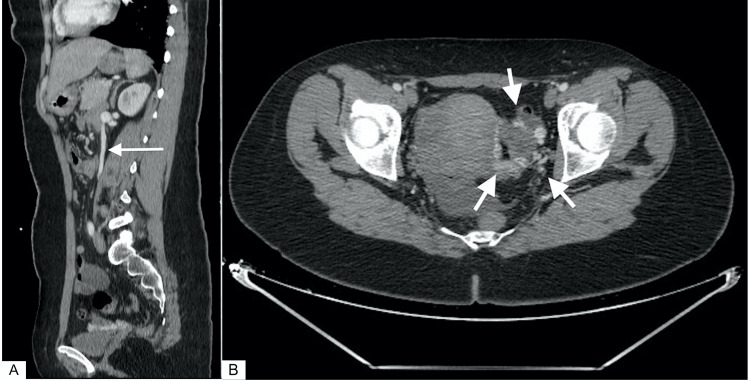
CT images of gonadal vein distension and pelvic congestion A: Dilatation of the left ovarian vein (white arrow) B: Pelvic venous circulation (white arrows)

CT scan also revealed dilatation of the LRV with a ratio of hilar to aorto-mesenteric diameters (A/B = 5) (Figure 2A), and narrowing of its segment trapped between the AA and the SMA (Figure 2B). On sagittal section, the angle between the AA (black arrow) and the SMA (red arrow) measured 22.7° (Figure 3). Imaging also found dilatation of the left ovarian vein (Figure 4A) and pelvic venous circulation (Figure 4B). These suggestive CT findings evoked the diagnosis of NCS. After multidisciplinary discussion, conservative treatment with a six-month follow-up period was decided given the moderate nature of the clinical symptomatology. 

## Discussion

The NCS refers to compression of the LRV as it passes through the aorto-mesenteric clamp. It is a rare entity, but probably underestimated. Its prevalence is higher in young subjects between 30 and 40 years of age, with a female predominance [2,5]. This was the case of this patient. The NCS can be classified into three types: anterior, posterior and mixed depending on the location of the LRV [6,7]. Anterior syndrome corresponds to compression of the LRV between the AA and the SMA. The posterior type, refers to compression of a retro-aortic LRV, betwixt the AA and the vertebral column [6].

NCS pathophysiology is still unclear. It may be caused by some anatomical variants [7,8] such as duplicity of the left renal vein, ectopic or kidneys fusion, etc. The female predominance could be explained by valvular alteration of the gonadal veins during pregnancy [9]. Furthermore, extrinsic LRV compressions can result in secondary NCS. Most secondary causes are para-aortic adenopathies; retroperitoneal tumors; and excessive fibrolymphatic tissue between the AA and the SMA [8]. Nevertheless, no association between appendicitis and NCS exists in the literature. In this case, the presence of these two pathologies was a coincidence.

Main symptoms of NCS are hematuria; chronic abdominal pain; dyspareunia and sensation of pelvic heaviness. No correlation exists between the intensity of the symptoms and the anatomical findings. Some subjects with marked compression of the LRV are completely asymptomatic [8]. Thus, the diagnosis of nutcracker relies on modern imaging techniques. When a patient presents with any of the cited symptoms, the diagnosis of NCS can be evoked. Actually, the initial examination used is Doppler ultrasound of the renal veins. It evaluates LRV flow and can reveal the compressive phenomenon [10]. CT angiography is the gold standard in the NCS diagnostic process [11]. Some authors have therefore attempted to establish diagnostic criteria, including Kim et al. [9]. They worked on four suggestive signs with different specificity degrees: On axial section, “Beak sign” was highly evocative of NCS with a specificity of 88.9%. It refers to the compression of the LRV and the narrowing of its aorto-mesenteric part. LRV diameter ratio (hilar/aorto-mesenteric) > 4.9 was the most specific imaging sign of NCS, with 100% specificity. On the sagittal section, an angle between the AA and the SMA (<41°) had a specificity of 55.6%. Finally, venous hyper-pressure in the LRV causes dilatation of the left ovarian vein and pelvic congestion. All this diagnostic criteria were present in this case.

NCS treatment can vary based on age and the severity of symptoms. Management options range from observation to nephrectomy. Conservative treatment is the rule for minimal, moderate or absent symptoms [12]. The possibility of spontaneous reduction of the compression must be considered before surgical therapy [13]. Several mechanisms explain the possibility of resolution of NCS symptoms after conservative treatment. Young individuals, through normal development, have an increase in intra-abdominal and retroperitoneal fibrous tissue at the origin of SMA, which can release LRV entrapment [14]. Furthermore, weight gain during conservative treatment was found to have beneficial effect on reducing LRV compression. Over a mean follow-up of 26 months, conservative treatment with emphasis on weight gain resolved NCS symptoms in 30% of patients [15]. Thus, the added retroperitoneal adipose tissue reduces LRV compression by opening the angle formed by the SMA and AA. Surgery is generally indicated in case of severe pain, massive hematuria or in case of ineffective conservative measures after six months follow-up for adults and 18 months for patients aged less than 18 years [4,16-17]. It could be a transposition of LRV, nephropexy, or nephrectomy [18]. Some other teams perform endovascular treatment using stents or embolizations techniques [19].

## Conclusions

NCS is a rare vascular disorder with various symptoms and unclear pathophysiology. Patients with NCS are likely to be non-diagnosed. It can evolve for years without being recognized. This syndrome must be considered in young patients presenting unexplained chronic abdominal or pelvic pain. CT angiography is the gold standard in establishing the diagnosis thanks to characteristic imaging features. This case presents an accidental discovery of NCS during an acute appendicitis episode. Treatment remains controversial. Management options range from therapeutic abstention with observation to surgical or endovascular approach. We hope through this case to highlight main clinical presentations and typical imaging findings of this uncommon syndrome. Finally, more researches are necessary for better understanding of NCS pathophysiology, its potential associations, and to establish management guidelines.

## References

[1] Cohen CT, Kirk S, Desai SB, Kukreja KU, Srivaths L (2021). Diagnosis, clinical characteristics, and treatment modalities of adolescent May-Thurner Syndrome-associated deep venous thrombosis. J Pediatr Hematol Oncol.

[2] Takezawa K, Nakazawa S, Yoneda S (2011). Renal autotransplantation for the treatment of nutcracker phenomenon which caused varicocele rupture: a case report. Hinyokika Kiyo.

[3] Grant J (1937). Method of Anatomy. https://uotechnology.edu.iq/dep/bme/english/Pages/Lectures/anatomy%20first%20course/Grant%27s%20Atlas%20of%20Anatomy.pdf.

[4] Ananthan K, Onida S, Davies AH (2017). Nutcracker syndrome: an update on current diagnostic criteria and management guidelines. Eur J Vasc Endovasc Surg.

[5] Andrianne R, Limet R, Waltregny D, de Leval J (2002). [Hematuria caused by nutcracker syndrome: peroperative confirmation of its presence]. Prog Urol.

[6] Haboussi MR, Tabakh H, Mouffak A (2021). [Nutcracker syndrome: a rare cause of abdominal pain in adults that shouldn't be ignored: a case report]. Pan Afr Med J.

[7] Orczyk K, Wysiadecki G, Majos A, Stefańczyk L, Topol M, Polguj M (2017). What each clinical anatomist has to know about left renal vein entrapment syndrome (nutcracker syndrome): a review of the most important findings. Biomed Res Int.

[8] Berthelot JM, Douane F, Maugars Y, Frampas E (2017). Le syndrome nutcracker: une cause rare de douleurs lombaires gauches mais aussi de souffrances pelviennes inexpliquées. Rev Rhum.

[9] Kim KW, Cho JY, Kim SH, Yoon JH, Kim DS, Chung JW, Park JH (2011). Diagnostic value of computed tomographic findings of nutcracker syndrome: correlation with renal venography and renocaval pressure gradients. Eur J Radiol.

[10] Calado R, Braz M, Lobo L, Simão C (2011). Síndrome de nutcracker: hematúria sem diagnóstico. Acta Med Port.

[11] Zucker EJ, Ganguli S, Ghoshhajra BB, Gupta R, Prabhakar AM (2016). Imaging of venous compression syndromes. Cardiovasc Diagn Ther.

[12] Avgerinos ED, McEnaney R, Chaer RA (2013). Surgical and endovascular interventions for nutcracker syndrome. Semin Vasc Surg.

[13] Shin JI, Lee JS, Kim MJ (2006). The prevalence, physical characteristics and diagnosis of nutcracker syndrome. Eur J Vasc Endovasc Surg.

[14] Shin JI, Baek SY, Lee JS, Kim MJ (2007). Follow-up and treatment of nutcracker syndrome. Ann Vasc Surg.

[15] Scultetus AH, Villavicencio JL, Gillespie DL (2001). The nutcracker syndrome: its role in the pelvic venous disorders. J Vasc Surg.

[16] Zhang H, Li M, Jin W, San P, Xu P, Pan S (2007). The left renal entrapment syndrome: diagnosis and treatment. Ann Vasc Surg.

[17] Kim JY, Joh JH, Choi HY, Do YS, Shin SW, Kim DI (2006). Transposition of the left renal vein in nutcracker syndrome. Eur J Vasc Endovasc Surg.

[18] Shin JI, Lee JS (2005). Nutcracker. Lancet.

[19] Chen S, Zhang H, Shi H, Tian L, Jin W, Li M (2011). Endovascular stenting for treatment of Nutcracker syndrome: report of 61 cases with long-term followup. J Urol.

